# Performance Assessment and Field Deployment of Carbon-Fiber-Reinforced Polymer (CFRP) Cables for Infrastructure Applications

**DOI:** 10.3390/polym18070811

**Published:** 2026-03-26

**Authors:** Sung-Jin Lee, Jongeok Lee, Woo-Tai Jung

**Affiliations:** Korea Institute of Civil Engineering and Building Technology, Goyang-si 10223, Republic of Korea; leesungjin@kict.re.kr (S.-J.L.); leeje@kict.re.kr (J.L.)

**Keywords:** carbon-fiber-reinforced polymer (CFRP) cables, anchorage system development, fatigue and relaxation performance, field application in civil infrastructure

## Abstract

Carbon-fiber-reinforced polymer (CFRP) cables have emerged as promising alternatives to conventional prestressing tendons because of their high tensile strength, excellent corrosion resistance, and low self-weight. Their use is particularly advantageous in infrastructure exposed to aggressive environments, such as chloride-induced corrosion, where improved durability and reduced maintenance are critically required. In this study, a 10 mm diameter round-bar-type CFRP cable was developed using a pultrusion process, and its applicability to structural systems was comprehensively evaluated through material testing and field implementation. Mechanical performance was assessed through tensile, relaxation, and fatigue tests. The developed CFRP cable exhibited an average tensile strength of 3019 MPa and an elastic modulus of 176.9 GPa, demonstrating mechanical properties comparable to or better than those of conventional prestressing tendons. The final relaxation ratio was measured as 2.25%, satisfying the low-relaxation criterion specified in KS D 7002. In the fatigue test, the cable sustained 2,000,000 loading cycles under a stress range corresponding to 60–66% of the ultimate tensile strength without fracture or significant stiffness degradation, confirming its excellent fatigue durability. In addition, the developed CFRP cable was implemented in a cable-net structure to verify its constructability and structural applicability in practice. The field application confirmed that the lightweight CFRP cable enabled convenient transportation and installation, while stable prestress introduction was achieved using the same tensioning procedure as that for conventional steel cable systems. The results demonstrate the integrated feasibility of the developed CFRP cable in terms of both material performance and practical structural application. This study provides experimental evidence supporting the structural use of CFRP tendons and offers a technical basis for the future development of design provisions and broader infrastructure applications.

## 1. Introduction

Reinforced concrete (RC) and prestressed concrete (PSC) structures have been widely adopted as fundamental structural systems in major infrastructure facilities, including bridges, tunnels, port facilities, and buildings. Despite their widespread use, their long-term structural performance is inherently vulnerable to deterioration arising from material heterogeneity, variations in construction quality, and environmental exposure such as chloride penetration and carbonation [1,2,3]. Among these factors, corrosion of prestressing tendons in PSC structures is particularly critical, as it leads to cross-sectional reduction, bond deterioration, and prestress loss, thereby adversely affecting load-carrying capacity and overall structural reliability [4,5]. This concern has been highlighted by several notable failures. For instance, the 2016 tendon rupture incident on the Seoul Inner Circular Expressway bridge illustrated the severe impact that corrosion of prestressing steel strands can exert on structural safety [6,7]. Similarly, the 2018 collapse of the Morandi Bridge in Italy raised worldwide awareness that corrosion-related deterioration and insufficient maintenance can critically threaten the long-term safety of PSC structures [8,9]. These incidents collectively indicate that securing the corrosion resistance and long-term durability of prestressing tendons is an essential prerequisite for ensuring the life-cycle safety and serviceability of infrastructure systems.

Although a variety of corrosion-protection techniques have been introduced to alleviate the deterioration of steel prestressing tendons, including surface coatings, increased concrete cover thickness, low-permeability concrete, and cathodic protection [10,11], their effectiveness is largely limited to delaying chloride ingress or suppressing electrochemical corrosion reactions. Likewise, protective treatments such as epoxy-coated reinforcement and galvanized steel have been widely implemented in practice [12,13]. Despite these efforts, such approaches do not fundamentally resolve the intrinsic corrosion vulnerability of steel. For this reason, growing attention has been paid to alternative structural materials that inherently possess non-corrosive and highly durable characteristics. Among them, fiber-reinforced polymer (FRP) materials have emerged as particularly attractive candidates for structural reinforcement and prestressing applications because of their high strength-to-weight ratio, excellent corrosion resistance, electrical insulation, and low thermal conductivity [14,15,16].

Since the industrial application of glass-fiber-reinforced polymer (GFRP) began in the 1940s, FRP has progressively developed into a high-performance structural material, particularly following advances in the aerospace industry during the 1960s, and has subsequently found broad applications in civil and building engineering [17,18]. Among its key advantages, FRP exhibits substantially lower self-weight and superior corrosion resistance compared with steel, rendering it particularly advantageous for infrastructure subjected to aggressive environments such as marine exposure and chloride attack [12]. Consequently, extensive research has focused on the application of non-corrosive FRP composites, including rebar, grids, composite piles, and prestressing tendons, in civil engineering structures. In particular, FRP tendons have attracted considerable attention as a promising alternative to conventional steel prestressing tendons because they can prevent cross-sectional loss caused by electrochemical corrosion and potentially extend maintenance intervals while reducing life-cycle cost (LCC) [19].

Furthermore, the recent global transition toward carbon neutrality has intensified the demand for low-carbon and high-durability construction materials. Steel and cement production are representative carbon-intensive industries, and considerable carbon emissions are also generated during the repeated repair and replacement of deteriorated structural materials over the service life of infrastructure. Accordingly, extending structural longevity through the adoption of durable alternative materials is increasingly recognized as an effective approach for reducing carbon emissions from a life-cycle perspective. In this regard, CFRP cables, which combine high tensile strength, a high elastic modulus, and excellent corrosion resistance, have emerged as particularly promising candidates for replacing conventional prestressing steel in infrastructure applications [20,21].

Research on FRP tendons was initiated at an early stage in Japan, where institutional and technical efforts first established the basis for their structural application. In the early 1990s, the Japan Society of Civil Engineers (JSCE) issued design and construction recommendations for continuous fiber-reinforced materials, thereby laying the groundwork for the use of FRP in structural systems [22]. Subsequent studies by Uomoto et al. examined the feasibility of FRP composites as concrete reinforcement and prestressing tendons, highlighting their potential advantages in terms of durability and constructability [23]. Japanese research efforts also extended to the long-term behavior of CFRP and AFRP tendons, including creep and relaxation characteristics, while more recent studies have focused on improving the anchorage performance of CFCC systems through pull-out testing [24].

In the United States, the structural application of FRP tendons was advanced through the publication of ACI 440.4R by ACI Committee 440, which provided design and construction recommendations for concrete structures prestressed with FRP tendons [25]. These recommendations were subsequently expanded for bridge applications through the AASHTO guide specifications [26] and NCHRP Report 907 [27]. Experimentally, Grace et al. and Abdelrahman and Rizkalla investigated the structural behavior of PSC beams and girders prestressed with CFRP tendons, thereby demonstrating their structural feasibility [28,29,30]. In addition, Saadatmanesh and Tannous evaluated the long-term durability of AFRP tendons with particular emphasis on creep and relaxation behavior [31], whereas Lees and Burgoyne examined the influence of tendon bond characteristics on the structural response of concrete beams and identified important design considerations [32].

In Europe, fib Bulletin 40 provided design recommendations for FRP reinforcement and prestressing tendons, thereby promoting international consensus on the structural application of FRP-based materials [33]. Bank et al. comprehensively assessed the manufacturing technology, economic viability, and structural behavior of FRP tendons and discussed their commercialization potential [34]. Subsequently, provisions related to FRP reinforcement were gradually incorporated into the revision of Eurocode 2 [35]. In parallel, European researchers have continued to investigate the anchorage behavior, long-term durability, and environmental resistance of CFRP tendons, thereby further substantiating their applicability in structural engineering practice.

Despite these advances, existing studies on CFRP tendons have predominantly focused on material characterization and member-level experimental validation, whereas integrated verification involving actual structural implementation remains relatively scarce. This gap is particularly pronounced in Korea, where structural application technologies for CFRP cables, including anchorage systems, remain at an early stage of development, and both experimental validation and field application cases are still scarce.

To address this gap, the present study developed a round-bar-type CFRP cable and experimentally evaluated its mechanical performance in terms of tensile behavior, relaxation response, and fatigue resistance. In addition, the developed CFRP cable system was applied to an actual structure to verify its constructability and practical structural feasibility. By presenting an integrated investigation that combines material performance evaluation with field implementation, this study seeks to provide a technical basis for the future establishment of design provisions and the broader adoption of CFRP tendons in engineering practice.

## 2. Development of CFRP Cable

Commercially available carbon-fiber prestressing tendons generally provide tensile strengths of more than 2000 MPa, while some products have been reported to exceed 2400 MPa. Based on this performance benchmark, the present study aimed to develop a CFRP cable with mechanical properties equivalent to or better than those of existing commercial products. In particular, considering its intended structural application, the key design objective was to secure adequate tensile strength and elastic modulus so that a prestressing force comparable to that of conventional prestressing steel strands could be introduced.

CFRP tendons can broadly be categorized into strand-type and rod-type systems depending on the manufacturing process adopted for the composite material. Strand-type tendons are fabricated by twisting multiple fiber bundles and therefore have a geometry similar to that of conventional steel strands. Despite this geometric advantage, their fabrication requires careful control of fiber alignment, precise resin impregnation, and uniform curing quality, resulting in a relatively complex manufacturing process. Furthermore, this type of configuration requires a dedicated anchorage system and involves relatively high technical demands in terms of process stability and quality uniformity [36,37].

Accordingly, in this study, a round-bar-type CFRP cable fabricated through a pultrusion-based process was adopted as the development target in order to secure manufacturing stability and quality uniformity. In the pultrusion process, continuous fiber bundles are first impregnated with resin, then passed through a shaping guide to form the desired cross-sectional geometry, and subsequently cured while traveling through a heated die. This process is advantageous in that it effectively preserves the axial alignment of fibers, thereby enabling the production of members with a constant cross section and stable mechanical properties. Furthermore, because it is inherently suitable for continuous manufacturing, pultrusion is regarded as an efficient and practical process for producing structural prestressing tendons.

The CFRP cable developed in this study was primarily composed of carbon fibers and epoxy resin, and its fiber volume fraction was designed to be approximately 80% to maximize axial tensile performance. A high fiber volume fraction is one of the principal parameters governing the tensile strength and elastic modulus of CFRP tendons, and is therefore essential for achieving the prestressing capacity required in prestressed concrete applications.

The diameter of the developed CFRP cable was determined to be 10 mm. This dimension was selected to allow the introduction of a prestressing force comparable to that of 1860 MPa-grade prestressing steel strands, which are widely used in conventional concrete structures. For the same nominal diameter, the target tensile strength of the CFRP cable was set at approximately 2800 MPa to ensure axial tensile performance equal to or greater than that of conventional steel strands. This target performance was established as a design criterion to compensate for the reduced cross-sectional area and to ensure the structural feasibility of replacing conventional steel prestressing tendons with the developed CFRP cable.

**Figure 1:**
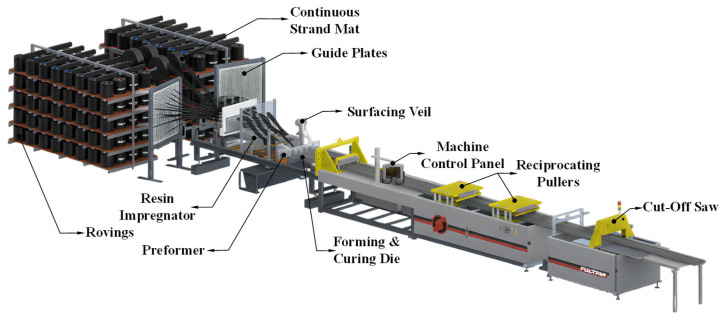
Manufacturing process of the CFRP cable by pultrusion.

Figure 1 presents the fabrication procedure of the CFRP cable based on the pultrusion process. In this process, carbon fiber bundles are first impregnated with resin and then passed through a shaping guide to form a circular cross section, followed by curing in a heated die. This continuous manufacturing procedure enables the production of CFRP cables with a uniform cross-sectional geometry and consistent mechanical properties.

## 3. Performance Evaluation of Developed CFRP Cable

### 3.1. Short-Term Performance Evaluation—Tensile Performance

As CFRP cables are the primary load-carrying elements used to introduce prestressing force into prestressed concrete structures, tensile performance evaluation is a fundamental prerequisite for verifying their structural reliability. In contrast to steel tendons, CFRP cables do not exhibit yielding behavior but instead remain linearly elastic until rupture, followed by brittle failure. For this reason, accurate evaluation of their mechanical properties, particularly ultimate tensile strength and rupture strain, is essential.

**Figure 2:**
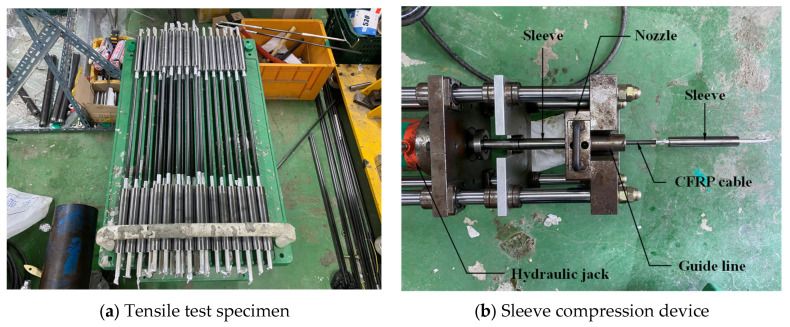
Fabrication of CFRP cable specimens.

The CFRP cable developed in this study was manufactured as a 10 mm-diameter round bar with a length of 1000 mm (Figure 2a). This geometric configuration was not directly compatible with the wedge-type anchorage system commonly used for conventional PS strands. To ensure stable mobilization of the tensile capacity of the CFRP cable, a dedicated compression-grip anchorage was therefore developed (Figure 2b) [38]. The anchorage system was designed to provide reliable transfer of axial tensile force from the carbon-fiber tendon, and its stress distribution was evaluated in advance by finite element analysis (FEA). In addition, a parametric study was conducted by varying the outer and inner diameters of the anchorage to ensure that failure occurred at the specimen mid-length rather than within the anchorage zone. Based on these analyses, the final dimensions of the compression-grip anchorage were determined to be approximately 26 mm in outer diameter and 11 mm in inner diameter.

**Figure 3:**
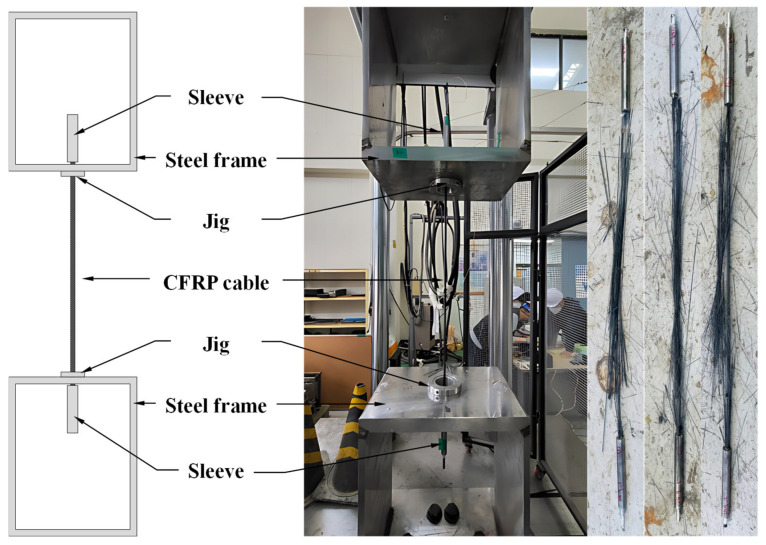
Tensile test setup for the CFRP cable specimen.

Tensile tests of the CFRP cable were carried out using a 1000 kN universal testing machine (UTM) (Figure 3). The tests were conducted in accordance with ISO 527 (KS M ISO 527) [39,40], which is widely used for evaluating the tensile properties of composite materials. Loading was applied under displacement control at a constant rate of 5 mm/min. To measure the axial strain response, a strain gauge was attached to the central gauge section of each specimen. In total, three specimens were tested.

**Figure 4:**
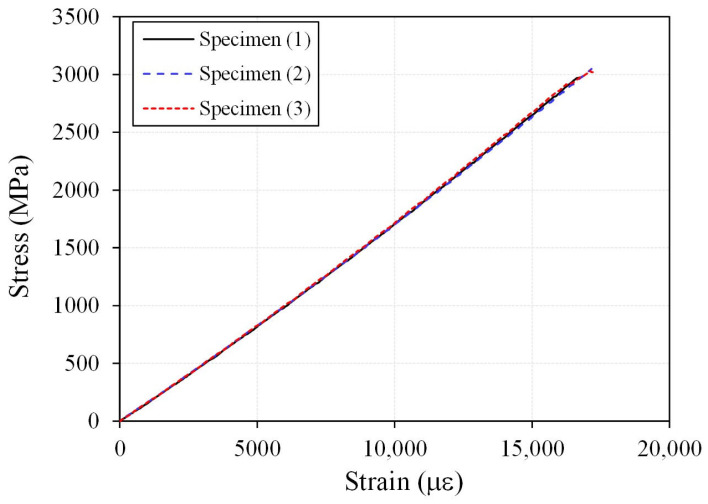
Stress–strain relationship from the tensile test.

The test results showed that, for all specimens, final failure occurred within the central gauge region rather than in the anchorage zone. This finding indicates that the developed compression-grip anchorage was able to reliably transfer the axial tensile force of the CFRP cable. In addition, the stress–strain response (Figure 4) remained linear up to the ultimate strength and was followed by sudden brittle failure, accompanied by an abrupt loss of load-carrying capacity without any discernible yielding region. This behavior is consistent with the typical linear-elastic brittle failure characteristics of CFRP materials.

Table 1 presents the tensile test results of the CFRP cable. The average tensile strength was 3019.2 MPa, and the measured tensile strength ranged from 2975.4 to 3049.0 MPa among the specimens. The average strain at peak was 17,065 με, and the average elastic modulus was 176.9 GPa. The coefficients of variation (CoV) for tensile strength, rupture strain, and elastic modulus were 0.0128, 0.0142, and 0.0051, respectively. These results indicate that the CFRP cable fabricated through the pultrusion process exhibited very limited variability among specimens, demonstrating a high level of uniformity and stability in its mechanical properties.

### 3.2. Long-Term Performance Evaluation—Relaxation

A relaxation test was performed to evaluate the long-term performance of the CFRP cable. Relaxation behavior refers to the time-dependent loss of prestressing force after the initial prestress has been applied, and is therefore considered a critical parameter affecting the long-term serviceability and structural reliability of prestressed concrete structures. Because the relaxation characteristics of a tendon are directly related to the reduction in effective prestress, they are regarded as one of the key evaluation items in the assessment of the long-term performance of prestressed structural systems.

**Figure 5:**
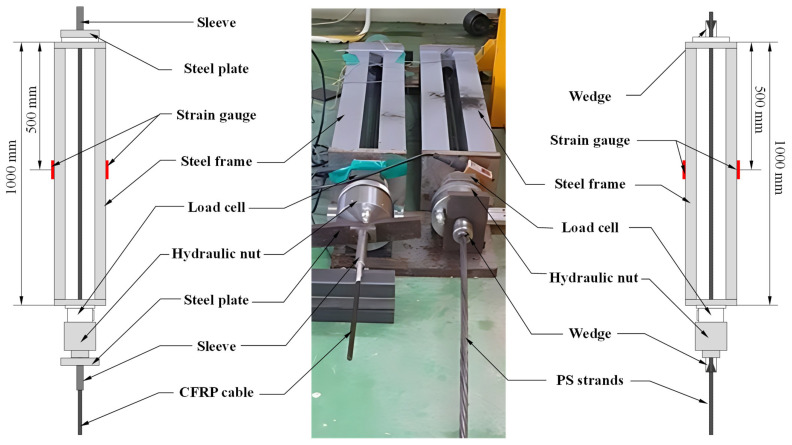
Experimental setup for the relaxation test of the CFRP cable and PS strand.

The relaxation test was performed under a constant strain condition, in which the time-dependent reduction in stress was measured while the imposed strain was maintained at a constant level. To preserve this constant strain state throughout the test duration, a restraining system with sufficiently high stiffness was required. Accordingly, a steel frame possessing substantially greater stiffness than the specimen was fabricated and used as the loading frame. In addition, a specially designed hydraulic nut device was employed to introduce the initial prestressing force in a stable manner and to maintain the constant strain condition during the entire test period. For comparative assessment of relaxation performance, a 1860 MPa-grade prestressing steel strand was selected as a reference specimen and tested under identical conditions. Furthermore, all tests were conducted in a temperature-controlled environment in order to minimize the influence of temperature fluctuations on the relaxation response. Figure 5 shows the relaxation test setup for the CFRP cable and the PS strand.

**Figure 6:**
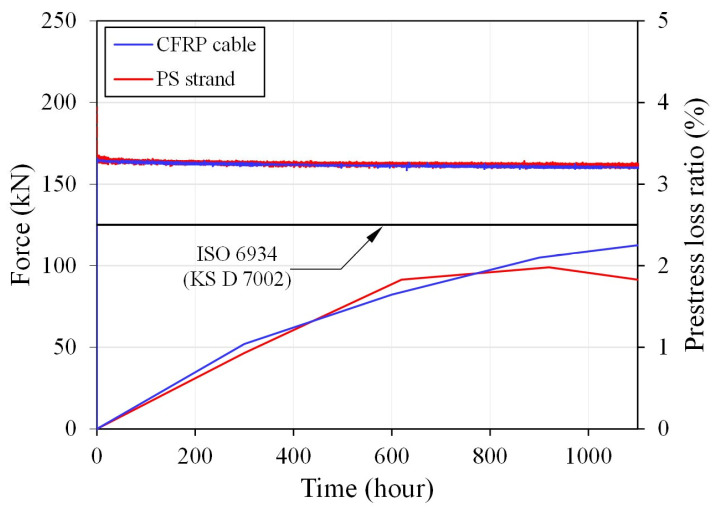
Force-time and prestress loss ratio-time relationships.

Figure 6 shows the force–time and prestress loss ratio-time relationships obtained from the relaxation test. At the start of the test, initial prestressing forces of 165.5 kN and 166.8 kN were introduced into the CFRP cable and the prestressing steel strand, respectively. The variation in prestressing force was then monitored for more than 1000 h under sustained loading conditions. Throughout the test period, both tendons exhibited a gradual decrease in prestressing force with time, and the overall relaxation behavior followed a similar trend.

At the end of the test, the prestressing force was measured as 160.0 kN for the CFRP cable and 161.8 kN for the prestressing steel strand. When the relaxation ratio was calculated directly from the initial and final prestressing forces, the resulting values exceeded the low-relaxation criterion of 2.5% specified in ISO 6934-4 (KS D 7002) [41,42]. However, this apparent exceedance was considered to reflect not only the intrinsic relaxation behavior of the tendons but also the effect of elastic deformation of the steel frame restraining the specimens during the test.

Accordingly, in order to evaluate the relaxation performance more accurately, strain gauges were installed at both ends of the steel frame to measure its deformation during the test. Based on the measured frame strain, the load variation associated with the axial stiffness of the frame was quantified and used to correct the measured prestress loss. After this correction, the final relaxation ratio of the CFRP cable was determined to be 2.25%, while that of the prestressing steel strand was 1.83% (Table 2). Both values satisfied the low-relaxation requirement of 2.5% or less specified in ISO 6934-4 and KS D 7002 [41,42]. These findings demonstrate that the developed CFRP cable possesses long-term stress retention capability comparable to that of conventional prestressing steel strands.

### 3.3. Long-Term Performance Evaluation—Fatigue

Fatigue performance of the CFRP cable is an important parameter directly related to the long-term durability and serviceability of prestressed concrete (PSC) structures subjected to cyclic loading. In infrastructure such as bridges, prestressing tendons experience repeated stress fluctuations due to traffic loads, wind actions, and temperature variations. Therefore, evaluation of their fatigue behavior is essential for ensuring structural reliability under long-term service conditions. In this study, the fatigue loading condition for the CFRP cable was determined by referring to the fatigue performance requirements for post-tensioning anchorages and couplers specified in the Korean Highway Bridge Design Code (Table 3) [43]. The code requires fatigue performance to be evaluated by applying cyclic stress within a specified range expressed as a percentage of the nominal ultimate strength of the tendon. Accordingly, the fatigue test was conducted under a stress range corresponding to 60–66% of the nominal ultimate tensile strength.

**Figure 7:**
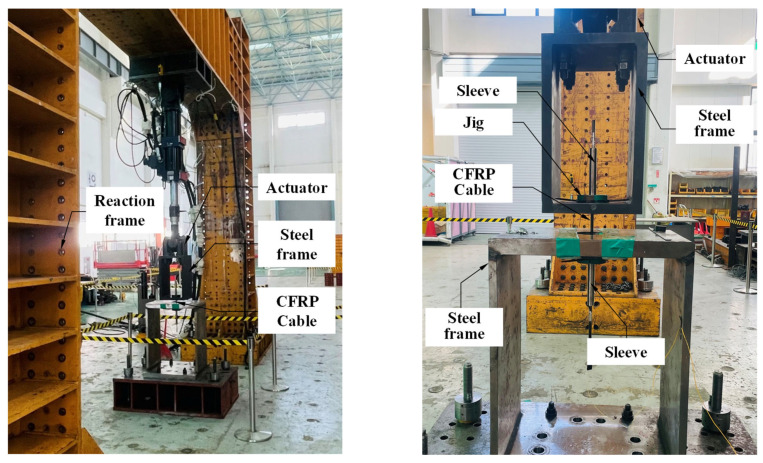
Experimental setup for the fatigue test.

As shown in Figure 7, the fatigue test was performed using a servo-hydraulic actuator, with the loading frequency maintained at a constant 3 Hz. Based on the target ultimate tensile strength of the developed CFRP cable, the cyclic load range was determined to be approximately 138–151.8 kN. A total of 500,000 loading cycles was initially applied to evaluate the early-stage fatigue behavior.

**Figure 8:**
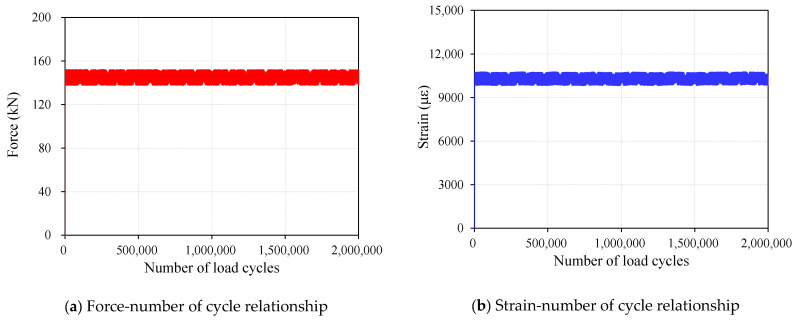
Fatigue response of CFRP cable during cyclic loading.

Following the application of 500,000 loading cycles, visual inspection confirmed that no surface defects, such as fiber rupture, resin debonding, or localized damage, were observed in the CFRP cable. In addition, analysis of the strain measurement data revealed no noticeable change in mechanical response during cyclic loading, including stiffness degradation or accumulation of residual deformation (Figure 8). These findings indicate that the CFRP cable maintained stable fatigue performance under the applied stress range.

**Figure 9:**
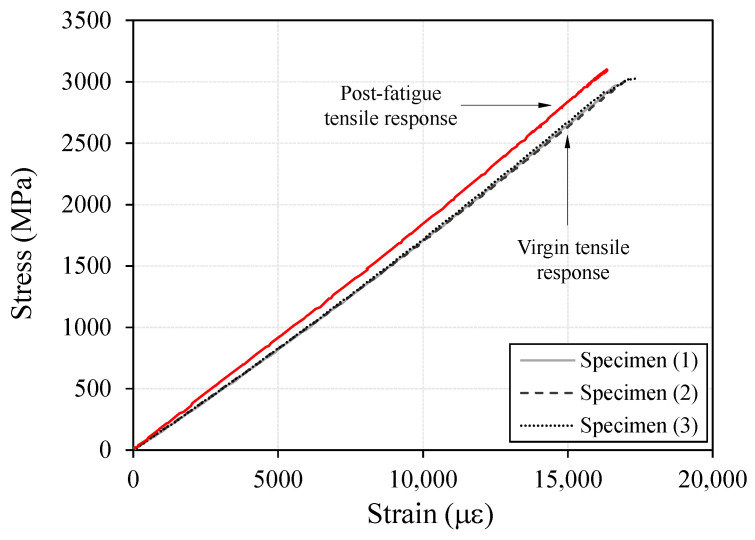
Comparison of tensile responses before and after fatigue loading.

To further assess durability under prolonged cyclic loading, an additional 1,500,000 cycles were applied following the initial 500,000 cycles, resulting in a total of 2,000,000 loading cycles. After completion of the fatigue loading, an additional tensile test was performed to evaluate the residual tensile performance of the CFRP cable. The test results confirmed that no slippage or localized damage occurred in the anchorage zone, and the stress–strain response remained linear elastic up to the point of failure (Figure 9). Final failure occurred at a stress level exceeding 3000 MPa, and the failure mode was identified as fiber-dominated brittle fracture. These findings indicate that the CFRP cable retained its tensile capacity even after extensive cyclic loading, suggesting that the developed system can provide adequate long-term fatigue durability and anchorage reliability for application in PSC structures.

## 4. Field Application of Developed CFRP Cable

The developed CFRP cable was intended for application in various infrastructure systems, including prestressed concrete (PSC) bridges. Because CFRP materials exhibit excellent corrosion resistance, they can be directly used in structures exposed to external environments without additional corrosion-protection measures, unlike conventional steel cables. This characteristic provides a distinct advantage for infrastructure located in aggressive environments, such as marine or chloride-laden conditions, by improving long-term durability and reducing maintenance requirements.

**Figure 10:**
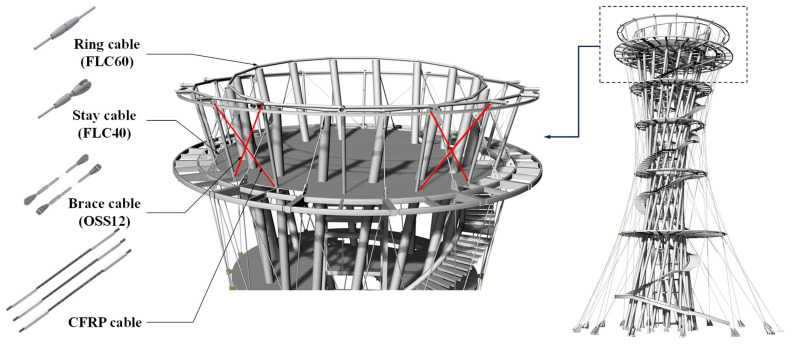
Structural system of the application site and installation locations of CFRP cables.

To verify the structural applicability of the developed CFRP cable, a field application was conducted on an actual structure. The application target was a cable-net structural system installed in an observation tower. In the original design, ∅12 OSS (Open Spiral Strand) cables and ∅40 and ∅60 FLC (Full Locked Coil) cables were used. In this study, four cables functioning as bracing members within the structural system were replaced with CFRP cables. Figure 10 shows the structural system of the application site and the installation locations of the CFRP cables, which are marked by red lines.

A comparison of the principal properties between the conventional ∅12 OSS cable and the developed CFRP cable indicated that the CFRP cable, despite its slightly smaller diameter of 10 mm, was capable of achieving a higher ultimate load due to its superior tensile strength. Moreover, the elastic modulus of the CFRP cable was approximately 170 GPa, demonstrating an axial stiffness comparable to that of the conventional steel cable. These results indicate that the developed CFRP cable provides sufficient structural performance for application in cable-net structural systems, as summarized in Table 4.

**Figure 11:**
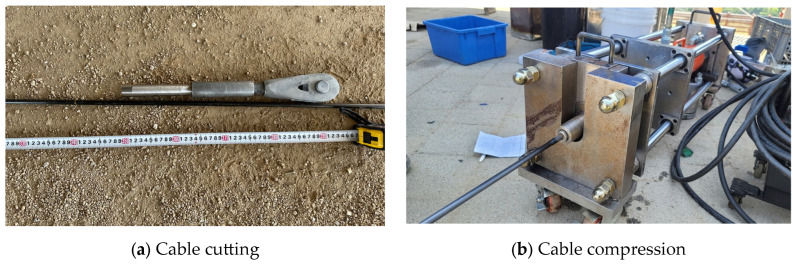

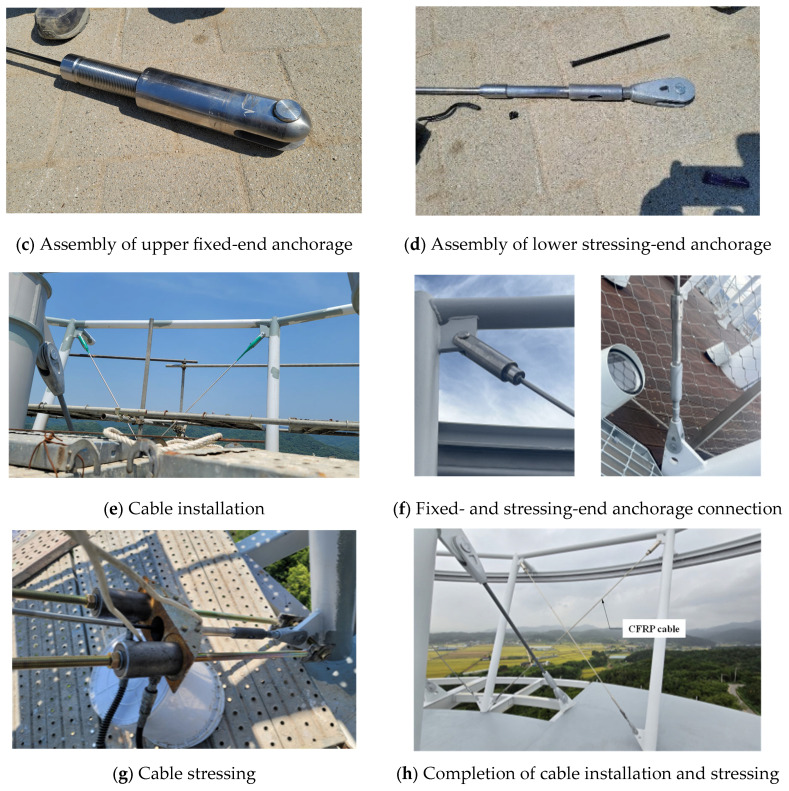
Fabrication of CFRP cables and assembly process of anchorages.

Each stage of the construction procedure is presented in Figure 11. The fabrication and installation of the CFRP cables were performed through a series of sequential operations.

For field application, the CFRP cables were cut to the lengths required by the design drawings, and an end-compression process was subsequently applied to ensure reliable load transfer between the cable and the anchorage device. During this process, the compression pressure and anchorage geometry were precisely controlled to secure adequate anchorage performance while minimizing fiber damage. The cable ends were then sequentially assembled to the upper fixed anchorage and the lower stressing anchorage, followed by installation in the structural system.

The stressing operation was conducted using the same procedure as that employed for conventional steel cable systems. Tension was introduced incrementally until the design prestress and target elongation were reached. During stressing, the load–displacement response and residual strain were monitored to confirm the proper introduction of the required prestressing force.

The field application demonstrated that the CFRP cable, owing to its lower unit weight than the steel cable, provided improved handling and constructability during transportation and installation. Moreover, the cable could be cut and assembled on site to satisfy the required pin-to-pin length, offering flexibility in accommodating length tolerances that may arise during construction. The installation process also confirmed that the same equipment and procedures used for conventional steel cable systems could be directly applied, enabling stable construction without additional installation equipment.

These results demonstrate that the developed CFRP cable is structurally compatible with existing steel cable systems and is suitable for practical application in real structures. In particular, its lightweight nature and corrosion resistance offer significant advantages in terms of long-term maintenance and suggest strong potential for broader application in various infrastructure systems, including cable-supported structures.

## 5. Conclusions

In this study, a carbon-fiber-reinforced polymer (CFRP) cable was developed as an alternative to conventional steel prestressing tendons to address corrosion-related deterioration and the resulting limitations in long-term durability. Its structural applicability was experimentally verified through mechanical performance evaluation and field implementation. The CFRP cable, fabricated using a pultrusion process, was assessed in terms of its material properties, long-term behavior, and practical applicability to an actual structure. The main conclusions of this study are as follows.

First, a 10 mm-diameter round-bar-type CFRP cable was successfully developed through pultrusion, achieving an average tensile strength of 3019 MPa and an average elastic modulus of 176.9 GPa. The coefficients of variation among the specimens were very low, indicating excellent material uniformity and stable mechanical performance.

Second, after correcting for the deformation of the steel frame, the final relaxation ratio of the CFRP cable was determined to be 2.25%, which satisfies the low-relaxation requirement specified in KS D 7002 and ISO 6934. In addition, no fracture or noticeable stiffness degradation was observed after 2,000,000 fatigue loading cycles, and subsequent tensile testing confirmed that the initial tensile performance was effectively retained. These results demonstrate the excellent long-term stress retention and fatigue durability of the developed CFRP cable.

Third, field implementation in an actual cable-net structure confirmed the practical feasibility of the developed CFRP cable. Owing to its low self-weight, the CFRP cable provided improved handling and constructability during transportation and installation. Moreover, stable introduction of the required prestress was achieved using the same stressing procedure as that employed for conventional steel cable systems. The ability to apply the cable without additional corrosion-protection treatment also suggests clear advantages in terms of long-term maintenance.

Overall, the developed CFRP cable was shown to possess sufficient mechanical performance, long-term durability, and practical constructability to serve as a viable substitute for conventional prestressing steel. This study is meaningful in that it presents an integrated verification of both material-level performance and real-structure application, thereby providing a technical basis for the future establishment of design provisions and the broader implementation of CFRP-based prestressing systems in infrastructure. Future studies should further address the accumulation of field application cases for various structural systems, optimization of anchorage system performance, durability assessment under long-term environmental exposure, and integration with structural health monitoring technologies for real-time performance evaluation. These findings collectively indicate that CFRP cables have strong potential as a promising structural alternative to conventional steel tendons in bridges and other infrastructure systems exposed to corrosive environments.

## Figures and Tables

**Table 1:** Tensile test results of CFRP cables.

Specimen ID	Ultimate Tensile Stress (MPa)	Strain at Ultimate Stress (με)	Elastic Modulus, *E* (GPa)
(1)	2975.4	16,789.1	177.2
(2)	3049.0	17,164.4	177.6
(3)	3033.1	17,242.2	175.9
Average	3019.2	17,065.3	176.9
Cov	0.0128	0.0142	0.0051

**Table 2:** Relaxation test results of the CFRP cable and PS strand.

Specimen Type	Initial Force, *F*_0_ (kN)	Final Force, *F_end_* (kN)	Frame-Deformation-Corrected Force, *F_corr_* (kN)	Relaxation Loss Ratio, *R_t_* (%)
CFRP cable	165.5	160.0	161.8	2.25
PS strand	166.8	161.8	158.8	1.83

**Table 3:** Fatigue performance of post-tensioning anchorages and couplers.

Conditions	Details
1	500,000 cycles at 60–66% of minimum ultimate strength
2	50 cycles at 40–80% of minimum ultimate strength

**Table 4:** Comparison of cable and anchorage specifications.

Items	Unit	Spiral Strand (OSS)	CFRP Cable
**Cable**			
(1) Diameter (D)	mm	12.0	10.0
(2) Sectional Area (A)	mm^2^	87.9	78.5
(3) Elastic Modulus (E)	GPa	160.0	170.0
(4) Axial Stiffness (EA)	MN	14.06	13.35
(5) Minimum Breaking Load, F_min_	kN	150.0	245.0
(6) Characteristic Breaking Load, F_uk_	kN	135.0	220.5
(7) Design Load, F_Rd_	kN	90.0	147.0
(8) Unit Weight (G)	Kg/m	0.7	-
(9) Corrosion Protection		Zn95Al5	
**Anchorage**			
(1) Threaded Stud		S355/S460	SM45C/STS329J2L
(2) Socket		S460N	
(3) Turnbuckle		S460	
(4) Pin		34CrNiMo6	

## Data Availability

The original contributions presented in this study are included in the article. Further inquiries can be directed to the corresponding author.
